# Inhibitory effects of components from root exudates of Welsh onion against root knot nematodes

**DOI:** 10.1371/journal.pone.0201471

**Published:** 2018-07-30

**Authors:** Tao Li, Hongyun Wang, Xiubo Xia, Shoujun Cao, Jiangang Yao, Lili Zhang

**Affiliations:** Yantai Agricultural Science Academy of Shandong Province, Yantai, Shandong, P. R. China; Fred Hutchinson Cancer Research Center, UNITED STATES

## Abstract

Root-knot nematodes (RKNs; *Meloidogyne* spp.) are obligate endoparasites that infect many crops and cause severe yield losses. In this research, we studied the effect of Welsh onion, grown as a companion plant, on the resistance of cucumber plants to RKN infection and analyzed the most abundant components of Welsh onion root exudates. The results showed that, when grown with Welsh onion as a companion plant, cucumber roots had 77.0% fewer root knots and egg masses than the control cucumber roots. Welsh onion root exudates were collected and extracted with chloroform, ethyl ether, *n*-butanol and ethyl acetate. High concentrations of the extracts from the Welsh onion root exudates decreased the hatchability of RKN eggs. In particular, the inhibitory effect of the *n*-butanol extract was significant and the hatchability of RKN eggs did not exceed 10%. Gas chromatographic–mass spectrometric analysis revealed that the most abundant component in the *n*-butanol extract was 4-hydroxy-benzeneethanol. Treatment with 1.2 mM 4-hydroxy-benzeneethanol decreased egg hatchability to 40%, whereas treatment with 9.6 mM or a higher concentration of 4-hydroxy-benzeneethanol decreased egg hatchability to less than 10%. In addition, 1.2 mM or a higher concentration of 4-hydroxy-benzeneethanol decreased the activity of the second-stage juvenile (J2). Higher 4-hydroxy-benzeneethanol concentrations (9.8 and 19.2 mM) were lethal to RKNs to some extent, with death rates greater than 50% at 48 h of treatment. The present results suggest that cultivation with Welsh onion as a companion plant may represent an alternative to the application of synthetic nematicides, with fewer side effects. We confirmed that 4-hydroxy-benzeneethanol is a natural effective nematicide.

## Introduction

As one of the most economically important vegetable crops, cucumber is popular worldwide for its flavor and nutritional value [1, 2]. However, diseases caused by plant pathogens, including root-knot nematodes (RKNs; *Meloidogyne* spp.), may result in stunted plant growth and significant yield loss [3, 4]. RKNs are obligate biotrophs capable of surviving in soil for many years and parasitize a variety of crops by stimulation of gall production [5, 6]. With crop production in greenhouses and the expanded use of continuous cropping systems, problems caused by RKNs are increasingly serious and control of RKNs in commercial farming operations is progressively more difficult [7].

Diverse management systems have been developed to prevent RKN infestations, including chemical control, soil heat treatment, flooding, biological control, and crop rotation, of which chemical control and soil heat treatment methods are widely used [8]. Although chemical control can considerably decrease the adverse effects of RKNs on vegetable yield, chemical nematacides are expensive, detrimental to the environment, or lead to the development of insecticide resistance. Consequently, there has been an increasing trend for implementation of agronomic practices as measures to control RKNs [9].

Three common agronomic practices have been developed for management of RKNs. One involves the incorporation of plant residues into the soil to function as green manure. Naz [10] observed that addition of purple pansy plants into the soil can decrease the root-knot index of tomato plants, promote tomato growth, and improve productivity. Monfort [11] confirmed that *Brassica* species produce glucosinolates, which show general biocidal effects, and incorporation of these species in green manure prior to the transplanting of vegetable crops may significantly decrease nematode populations without adversely affecting the growth and yield of the subsequent vegetable crop. Thus, green manure may suppress the RKNs and other soil-borne plant pathogens by influencing the antagonism and competition among soil microbes. An additional agronomic practice used to control RKNs is associated with the planting system. For example, interplanting tomato with garlic [12], or Spanish clover and *Leucaena* species, in a crop rotation system effectively controls RKNs in the field, possibly because of plant secretion characteristics [13]. The third agronomic practice involves the use of extracts from different plant parts. Olabiyi [14] observed that aqueous extracts from plant roots were effective in decreasing the abundance of RKNs in the soil, which helped to protect tomato plants from RKN infection. Black pepper leaf extracts are reported to inhibit nematode growth and development in a dose-dependent manner. These extracts penetrate the nematode egg mass matrix and significantly restrict egg hatching as well as kill J2 (i.e., the second-stage juveniles) and enhance plant growth [15]. Moreover, extracts from neem [16], marigold [17], and mint [18] are directly lethal to nematodes.

Plant-synthesized compounds in the rhizosphere originating from root exudates or sites of previous nematode penetration can influence nematode behavior. A number of compounds released by plants, of which some are present in root exudates, either attract nematodes to the roots, or result in repellence, motility inhibition, or even death of nematodes. For example, analysis of tomato root exudates indicates that four compounds, namely 2,6-di-*tert*-butyl-*p*-cresol, *L*-ascorbyl 2,6-dipalmitate, dibutyl phthalate, and dimethyl phthalate, were capable of suppressing *Meloidogyne incognita* eggs from hatching and were associated with increased J2 mortality [19]. Dutta [20] observed that small lipophilic molecules present in the root exudates of tomato and rice negatively affect *Meloidogyne* spp. mobility.

Welsh onion (*Allium fistulosum* L.) belongs to the Amaryllidaceae family. Previous studies revealed that when grown as a companion plant or as part of a crop rotation, Welsh onion was not infected by RKNs [21]. In addition, Welsh onion lessens the severity of RKN-induced damages to the companion plants or the subsequent crop in a crop rotation. Therefore, Welsh onion may have a unique protective mechanism against RKNs. We previously observed that the use of Welsh onion as a companion plant can control RKNs [22]. Elucidation of the mechanism underlying the inhibitory effect of Welsh onion extract components on RKNs may have important implications for crop production. In the present study, we prepared four extracts of Welsh onion root exudates and assessed their influence on hatching of *Meloidogyne* egg. We also screened the dominant compounds in the root exudate and analyzed their effects on egg hatching and growth of J2.

## Materials and methods

### Materials and growth conditions

The entire experiment was performed from 2013 to 2017 in the Yantai Academy of Agricultural Science, Shandong, China. The Welsh onion (*Allium fistulosum* L. var. *giganteum* Makino) cultivar ‘Tie Gan’ was used in the study. The cucumber (*Cucumis sativus* L.) cultivar ‘YU XIU’ was bred by the Yantai Academy of Agricultural Science, Shandong Province, China. The second-stage juvenile (J2) and eggs of the RKN *Meloidogyne incognita* used for inoculations were provided by the Key Laboratory of Plant Protection of Shandong Agricultural University, Shandong Province, China. Suspensions of eggs containing 10,000 eggs/mL and of J2 containing 6000 J2/mL were prepared.

Cucumber and Welsh onion seeds were sown in trays containing turf soil and incubated under a 16-h/8-h (light/dark) photoperiod and corresponding 25°C/18°C temperature regime in a phytotron. When the cucumber seedlings had three to four true-leaves and the Welsh onion seedlings were about 20 cm tall, the plants were transferred to a greenhouse.

### Greenhouse growth experiment

The greenhouse had been used to cultivate tomato and cucumber for 5 years, during which severe RKN infection occurred. We first ploughed and cultivated the experimental plot. To account for the possible uneven distribution of RKNs in the soil, we established three test plots of about 20 m^2^ randomly for each treatment. About 90 cucumber plants were cultivated in each test plot. Two treatments (S1 Fig) were applied, namely cucumber plants cultivated in monoculture (with 40 cm spacing between plants; CK) and cucumber plants grown with Welsh onion as companion plants (i.e., each cucumber was planted with two Welsh onion seedlings on both sides, with 8 cm spacing between cucumber and welsh onion plants; T). The two treatments were applied in separate 20-m^2^ test plots each planted with about 90 cucumber and 180 Welsh onion plants. The prophase fruit yield corresponded to the first 25 days after the early fruit stage. The fruit yield of this stage indicated the early growth of the plant. Whereas, the total fruit yield refers to the entire fruit production period. The experimental results presented for each treatment are the averages for the three test plots.

### Assessment of populations of RKNs and eggs

End of the cultivation, cucumber roots were washed free of soil and weighed. The total number of egg masses on the whole root system was counted and 10 egg masses were randomly selected. The egg masses were individually dissolved in 0.5 mL concentrated NaClO to release the eggs. Dilutions were prepared and the number of eggs per egg mass was counted; the mean number for the 10 egg masses was calculated. At least three repetitions were performed for each treatment. For estimation of the total nematode population, the roots were stained with the acid fuchsin method described by Byrd et al. [23]. The number of J2 were observed and counted under the microscope (Motic A310, Xiamen, China). An individual juvenile female contains about 200–300 eggs, so the number of female juveniles was estimated from the number of eggs.

### Collection of Welsh onion root exudates

Welsh onion root exudates were collected by cultivation in nutrient solution in a bioclean room using the method described by Wang et al. [24]. The collecting device consisted of an incubator and a small oxygen pump (S2 Fig, Utility Model Patent of the People’s Republic of China No. ZL 2016 2 1037421.2). To more realistically simulate the onion root growth environment, ceramsite was used as the substrate to cultivate Welsh onion because of its stability and it is inert. Prior to the experiment, the ceramsite was washed to remove surface impurities, after which it was soaked for 12 h in distilled water and then air dried.

When about 25 cm in height, Welsh onion plants were fixed to the root secretion-collecting device. Hoagland solution (15L) [25] [0.75 mM K_2_SO_4_, 0.65 mM MgSO_4_, 0.1 mM KCl, 2.0 mM Ca(NO_3_)_2_, 0.25 mM KH_2_PO_4_, 0.1 mM Fe-EDTA, 0.01 mM H_3_BO_3_, 5 × 10^−6^ mM (NH_4_)_6_Mo_7_O_24_, 0.01 mM MnSO_4_, 1 × 10^−4^ mM CuSO_4_, and 1 × 10^−3^ mM ZnSO_4_] with 25% ceramsite was placed in the incubator. The Welsh onion plants were fixed at the top of the incubator, with all roots submerged in the solution. The experiment was conducted for 60 days, during which the nutrient solution was collected and refrigerated after 30 days. Fresh nutrient solution was added to the incubator and collected upon completion of the experiment. The roots of the Welsh onion plants were weighed at the end of the experiment. The nutrient solutions collected after 30 and 60 days were combined. A vacuum evaporation system (RE-52AA, Yarong, Shanghai, China) was used to concentrate the nutrient solution to 2.0 g/mL (each milliliter of nutrient solution contained the exudates of 2.0 g Welsh onion roots), and stored at 4°C.

### Effect of Welsh onion root exudate components on the hatching of RKN eggs

A 100-mL sample of the concentrated (2.0 g/mL) Welsh onion root exudate solution was treated three times with ethyl ether, ethyl acetate, chloroform, and *n*-butanol (ratio of root exudate to organic solvent was 2:1, v/v). The extracts were dried with anhydrous sodium sulfate and a reduced-pressure rotary evaporation apparatus. The residues were redissolved in 5 mL methanol and stored at −20°C [26].

A 3 mL sample was taken from each of the four extracts and, after the methanol had evaporated completely, 3 mL sterile water was added for use. As the control, 3 mL methanol was placed in a centrifuge tube and evaporated completely, and then 3 mL sterile water was added to the tube. Each well of a 96-well flat cell-culture plate was filled with 20 μL eggs suspension (about 200 eggs), an aliquot of one of the four onion root extracts (10, 20, 50, 100, 150, or 200 μL), and sterile distilled water to make a final volume of 300 μL. All extract volumes were tested in triplicate. The cell-culture plates were incubated at 26°C in a biochemical incubator, and the hatching of eggs was examined every 2 or 3 days with an inverted biological microscope (Olympus, IX51-12PH, Tokyo, Japan).

### Separation and GC-MS analysis of the active ingredients in Welsh onion root exudates

Extract composition was analyzed by gas chromatography and mass spectrometry (GC-MS). The GC conditions were as follows: column: Rtx-WAX elastic quartz capillary tube chromatographic column (30 m × 0.35 mm × 0.35 μm; Restek Corporation, Bellefonte, PA, USA); injection port temperature: 250°C; column temperature program: 100°C for 2 min, increasing to 250°C at 10°C /min, and maintained at 250°C for 10 min; carrier gas: high purity helium (99.999%); flow rate: 1.0 mL/min; sample volume: 1.0 μL; and diversion ratio: 50. The MS conditions were as follows: ion source temperature: 230°C; electron energy: 70 eV; interface temperature: 250°C; electron multiplier voltage: 1,588 V; ion monitoring mode: scan mode; ion monitoring scope: 50 to 500; and solvent delay: 2.5 min.

### Effect of exogenous 4-hydroxy-benzeneethanol on the hatching of RKN eggs and J2 nematode survival

4-Hydroxy-benzeneethanol (0.53 g; Sigma, St Louis, MO, USA) was diluted in 200 μL ethyl alcohol, and then was added to 100 mL sterile water to generate a 38.4 mM stock solution. The solution was further diluted with sterile water to produce the treatment concentrations. The control solution was 200 μL ethyl alcohol added to 100 mL sterile water, which was further diluted with sterile water to correspond with the treatment concentrations of 4-hydroxy-benzeneethanol solutions.

Each well of a 96-well flat cell culture plate was filled with 20 μL eggs suspension (about 200 eggs), different concentrations of 4-hydroxy-benzeneethanol solutions (0.3, 0.6, 1.2, 2.4, 4.8, 9.6, and 19.2 mM), and sterile distilled water to make a final volume of 300 μL. All 4-hydroxy-benzeneethanol concentrations were tested in triplicate. Sterile distilled water was used as the control solution. The J2 death rate in response to 4-hydroxy-benzeneethanol treatment was similarly determined, with each well containing 20 μL J2 suspension (about 120 juveniles) instead of eggs. The cell-culture plates were incubated at 26°C in a biochemical incubator. The eggs and J2 nematodes were examined with an IX51-12PH inverted biological microscope. The hatching of eggs was examined every 2 or 3 days, and the J2 mortality rate was assessed by the needle stimulus method at 8, 24, and 48 h [27].

The egg hatching rate and J2 death rate were calculated as follows:

Hatching rate = number of hatched eggs/total number of eggs×100%

Death rate = number of dead J2 /total number of J2×100%

### Data analysis

Data underwent an analysis of variance using SPSS software (version 14.0) (SPSS Inc., Chicago, IL, USA). Treatment means were analyzed by least significant difference tests at the *P* ≤ 0.05 significance level.

## Results

### Effect of Welsh onion as a companion plant on RKN infections of cucumber plants in the greenhouse

**Fig 1:**
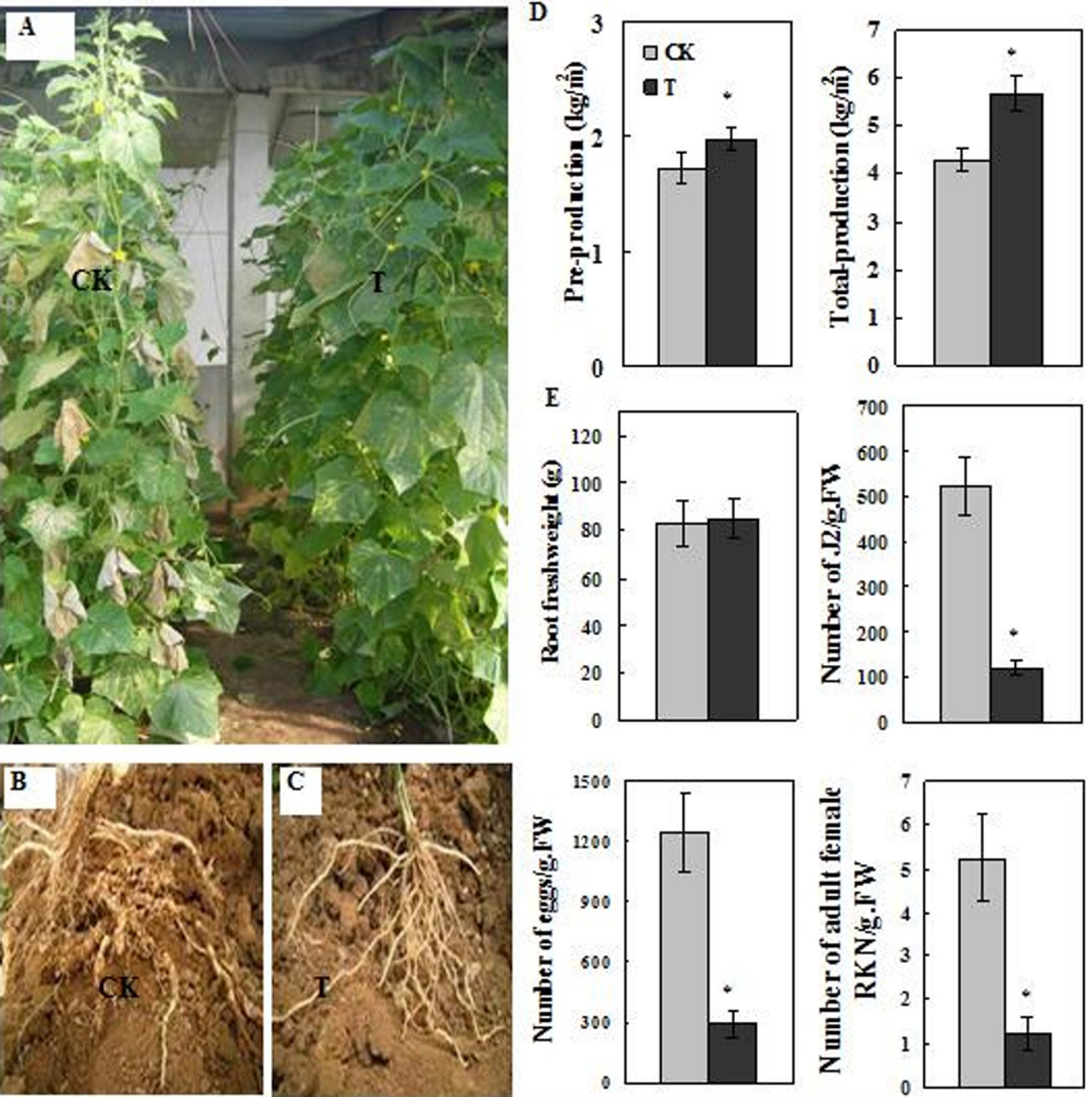
Effects of Welsh onion as a companion plant on root-knot nematode (RKN) infections of cucumber plants. (A) Cucumber plants cultivated in monoculture (CK) or with Welsh onion (T). (B) Roots of CK cucumber plants. (C) Roots of T cucumber plants. (D) Cucumber production during the preproduction and total production stages. (E) Root fresh weight and the number of RKNs, eggs, and adult female RKNs in cucumber roots. Each point is the average of three individual experiments. Error bars represent the standard deviation. * indicates a significant difference (P ≤ 0.05).

The cucumber monoculture (CK) exhibited typical symptoms of RKN infections. Specifically, the leaves were yellow and wilted. In contrast, the cucumber plants cultivated in combination with Welsh onion plants (T) showed superior growth and a healthy appearance (Fig 1A). Compared with the roots of the CK plants (Fig 1B), the root system of T plants had fewer galls and egg masses (Fig 1C). The roots of Welsh onion also had no galls. These results indicated that the inclusion of Welsh onion as a companion plant increased the RKN resistance of cucumber plants. The prophase fruit yield of CK and T plants was 1.7 ± 0.6 kg/m^2^ and 2.0 ± 0.4 kg/m^2^, respectively (i.e., 14.5% higher for T plants). The total fruit yield of CK and T plants was 4.3 ± 1.2 kg/m^2^ and 5.7 ± 1.1 kg/m^2^, respectively (i.e., 32.4% higher for T plants) (Fig 1D). The root fresh weight was higher for T plants than for CK plants, but not significantly. We observed 522.6 ± 36.4 J2 nematodes, 1,245.8 ± 58.1 eggs, and 4.2 ± 0.9 to 6.3 ± 1.1 female adult RKNs per gram fresh roots for CK plants, which was significantly higher than the 121.5 ± 23.4 J2 nematodes, 290.4 ± 38.1 eggs, and 1.3 ± 0.2 female adult RKNs per gram fresh roots recorded for T plants. The roots of T plants had 77.0% fewer RKNs and eggs than the roots of CK plants (Fig 1E). These results implied that the Welsh onion root exudates inhibited RKN growth and activity.

### Effect of extracts of Welsh onion root exudates on the hatching of RKN eggs

**Fig 2:**
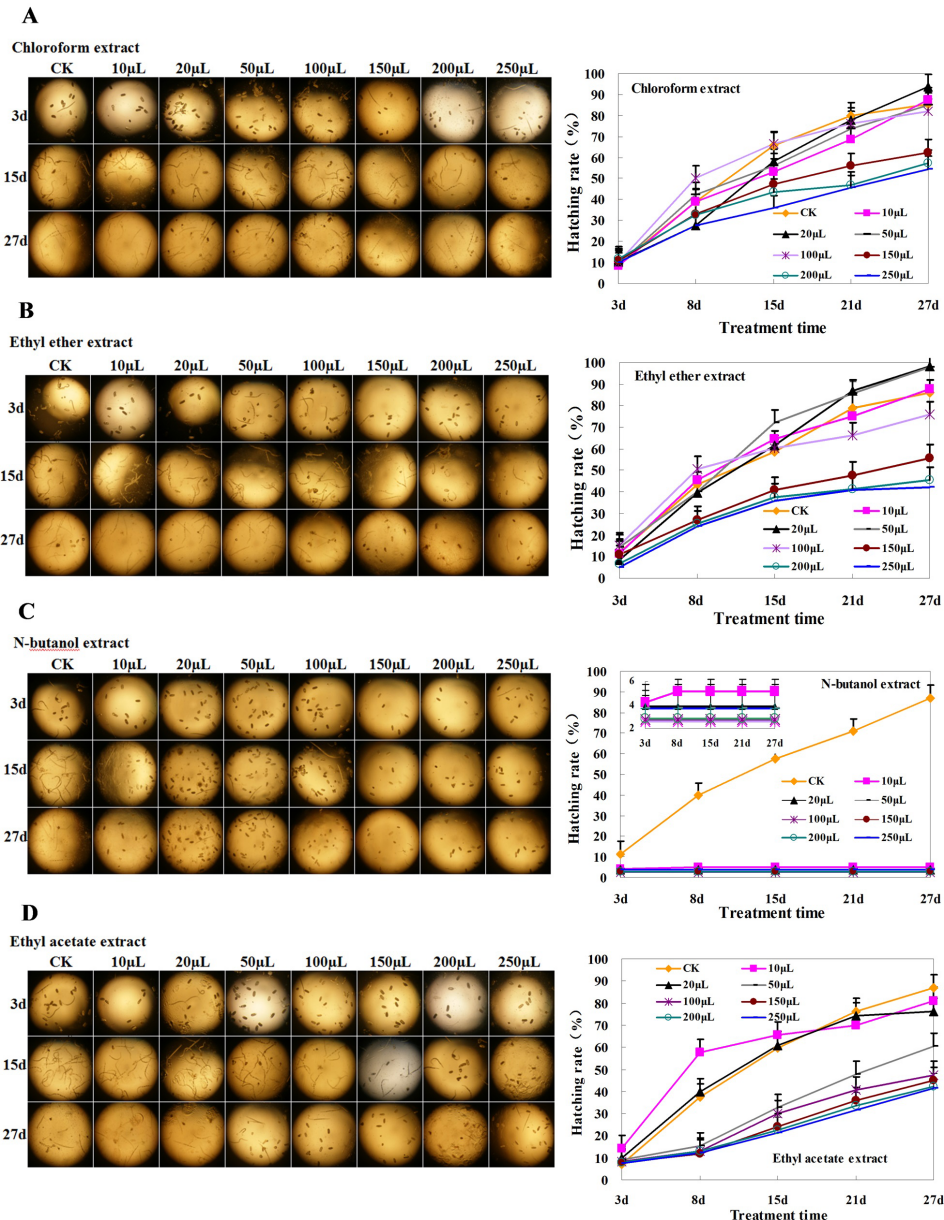
Hatching rate of RKN eggs in the presence of different concentrations of chloroform, ethyl ether, *n*-butanol, or ethyl acetate extracts of Welsh onion root exudates. (A) Chloroform extract, (B) ethyl ether extract, (C) *n*-butanol extract, and (D) ethyl acetate extract. The labels (i.e., 10 μL, 20 μL) above each figure indicate the volume of the four extracts used, CK was the control, in which sterilized water was used instead of an extract. Each point is the average of three individual experiments. Error bars represent the standard deviation.

The hatchability of eggs under treatment with the four root exudates extract increased over time, and egg hatchability generally decreased with increasing extract concentrations (Fig 2). The final egg hatchability of the control treatment (CK) in the four treatments ranged from 80% to 90%. In chloroform and ethyl ether extract treatments, an inhibitory effect was not obvious at the four lowest concentrations (10, 20, 50, and 100 μL), with egg hatchability ranging from 75 to 98%. The higher concentrations (150, 200, and 250 μL) of extracts resulted in egg hatchability ranging from 42% to 62% (Fig 2A and 2B). These results indicated that components of the chloroform and ethyl ether extracts were capable of inhibiting the hatching of RKN eggs at a sufficiently high concentration. The ethyl acetate extract was more effective than the chloroform and ethyl ether extracts for inhibiting the hatching of eggs. Volumes of 50 μL or higher decreased the egg hatching percentage to 40% to 50% (Fig 2D).

Notably, the *n*-butanol extract also significantly inhibited the hatching of RKN eggs (Fig 2C). Although some eggs were able to hatch in the first 5 days at the lowest volume (10 μL), the hatchability of eggs at the other volumes remained low over time. These results implied that components of the *n*-butanol extract quickly inhibited the hatching of RKN eggs.

### Welsh onion root exudate contents of the four extracts

High concentrations of the four Welsh onion root extracts inhibited the hatching of RKN eggs to some extent. The components of the four root exudate extracts were analyzed by GC-MS.

**Fig 3:**
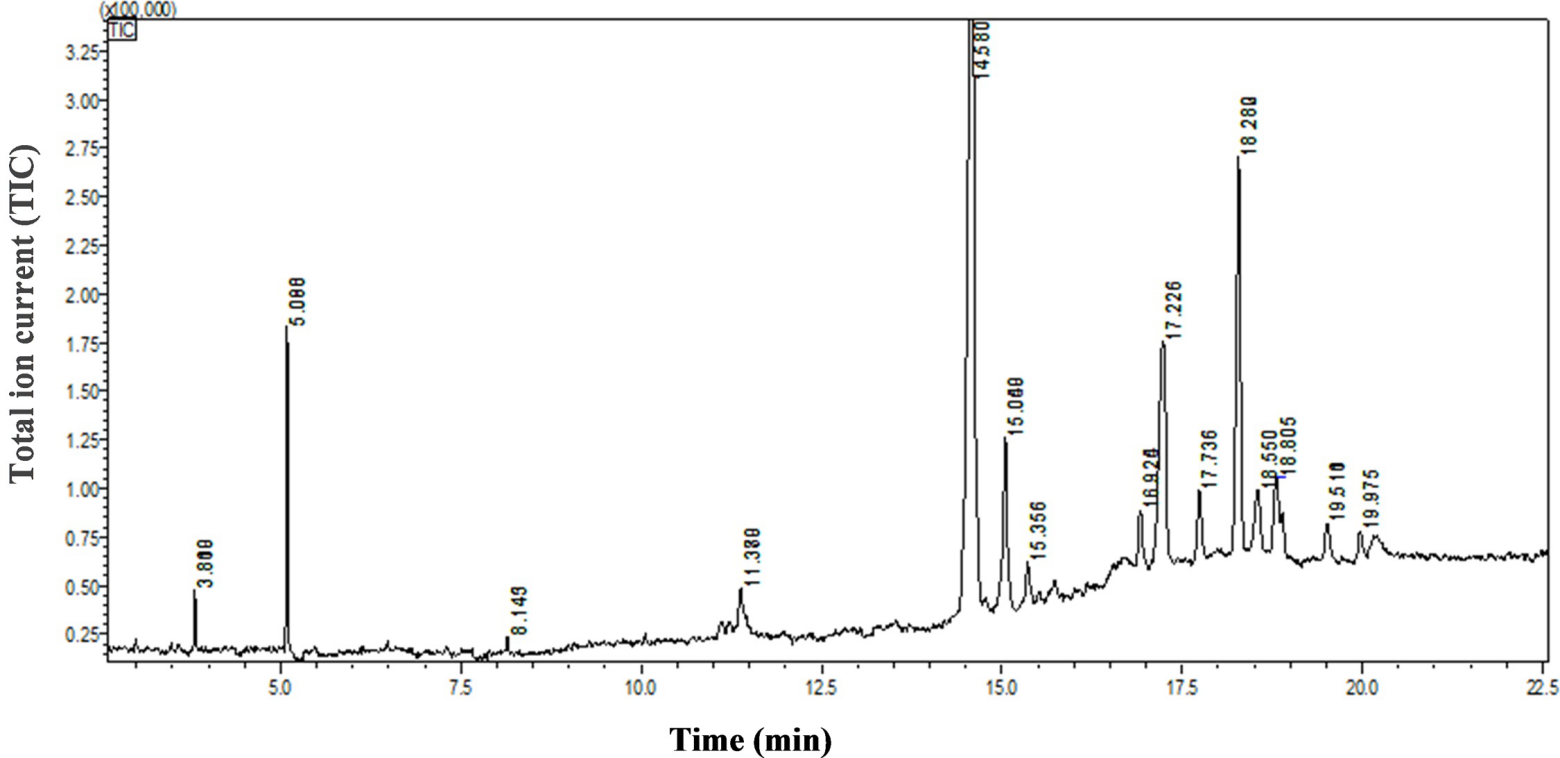
GC-MS spectrum for the *n*-butanol extract of Welsh onion root exudates.

**Fig 4:**
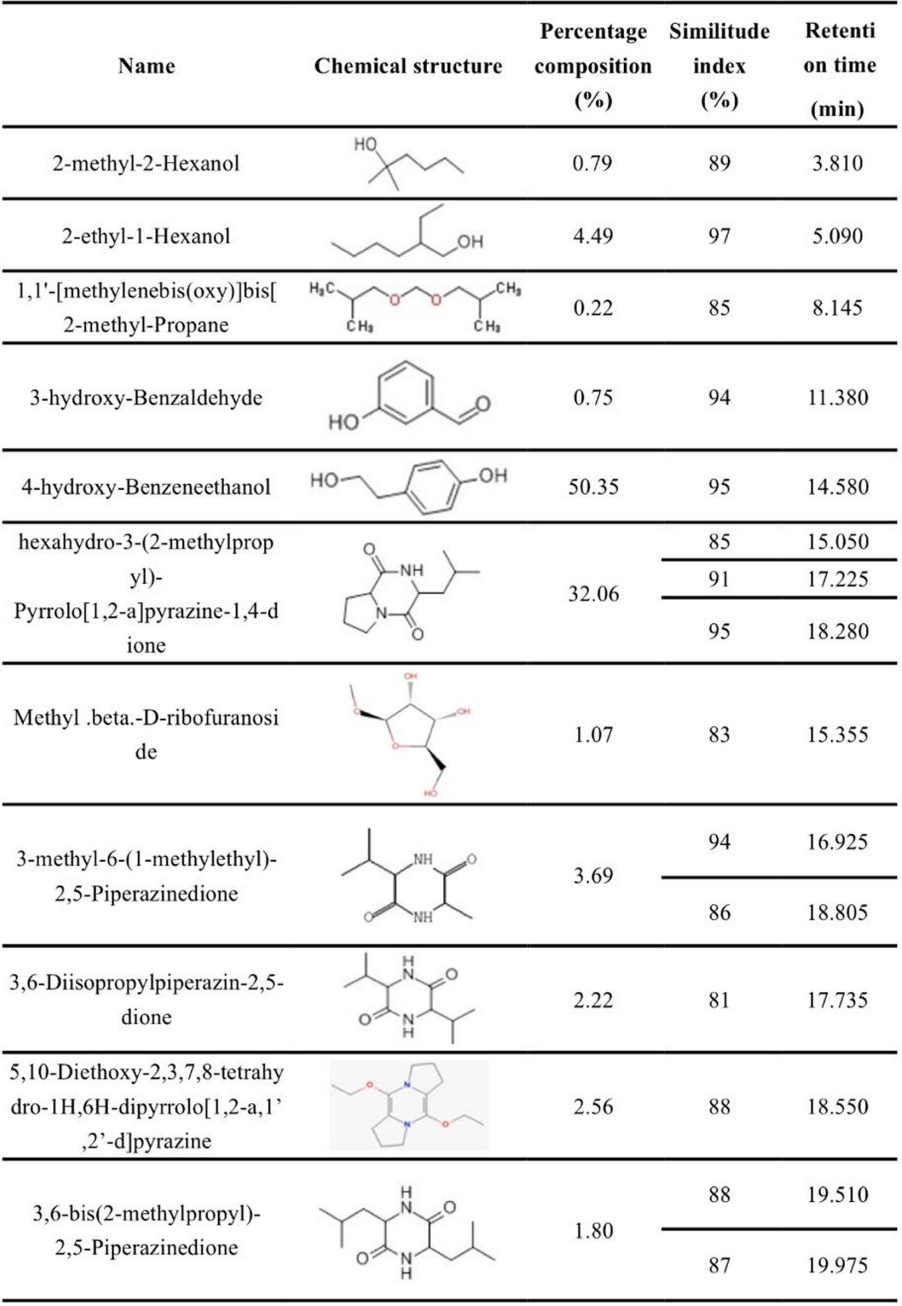
The main compositions of Welsh onion root exudates extracted with *n*-butanol.

The *n*-butanol extract comprised 11 components. The two main components were 4-hydroxy-benzeneethanol (50.35%) and proline cyclodipeptide (32.06%), which were separated at 14.580 min and 18.280 min respectively. The other components were present at low concentrations (Figs 3 and 4). The chloroform extract consisted of three components: cyclic-phenylalanine-proline (72.48%) dihydroergotamine (21.27%) and diacetone alcohol (6.25%) (Panel A in S3 Fig and S1 Table). The most abundant components in the ethyl ether extract were formaldehyde tyramine (67.90%), 2-methyl-2-hexoxyl (18.47%), and fast blue BB salt (13.63%) (Panel B in S3 Fig and S2 Table). The two components of the ethyl acetate extract were butyl acetate (84.73%), methyl ketone alcohol (15.27%) (Panel C in S3 Fig and S3 Table).

### Effect of exogenous 4-hydroxy-benzeneethanol on the hatching of RKN eggs and J2 survival

**Fig 5:**
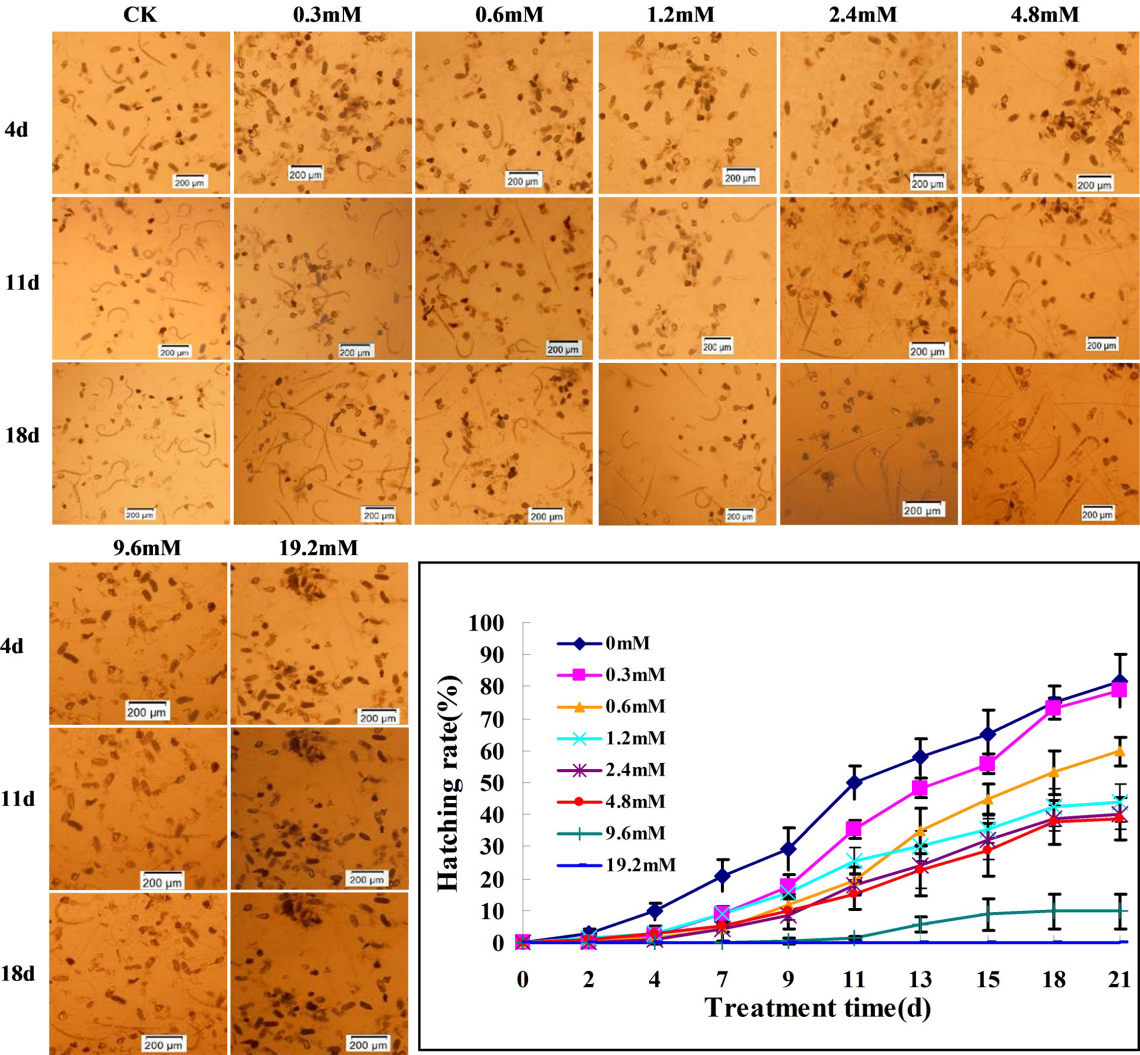
Effect of different concentrations of 4-hydroxy-benzeneethanol on the hatching of RKN eggs. Each point is the average of three individual experiments. Error bars represent the standard deviation.

The *n*-butanol root extract inhibited the hatching of RKN eggs, and one of the dominant components of the extract was 4-hydroxy-benzeneethanol. A subsequent experiment revealed that 4-hydroxy-benzeneethanol inhibited RKN egg hatching, and the inhibition of hatchability increased with elevation in 4-hydroxy-benzeneethanol concentration. At 1.2 mM and higher, egg hatchability was less than 50%. At 9.6 mM egg hatchability was less than 10%, and at 19.2 mM almost no eggs were capable of hatching (Fig 5).

**Fig 6:**
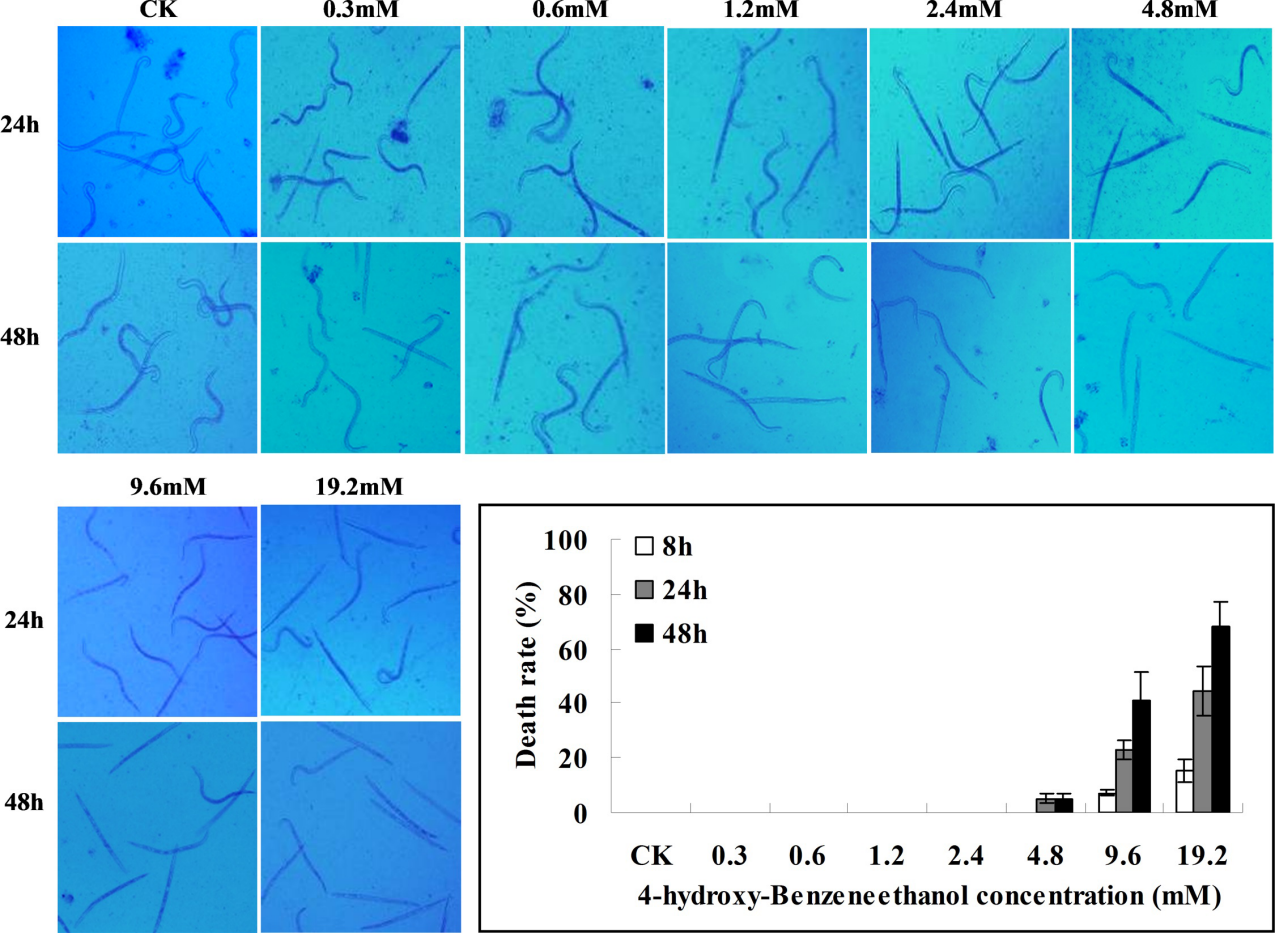
Effect of different concentrations of 4-hydroxy-benzeneethanol on RKN growth and survival. Each point is the average of three individual experiments. Error bars represent the standard deviation.

Investigation of J2 nematode response to different concentrations of 4-hydroxy-benzeneethanol indicated that 48-h treatment with 0.3 or 0.6 mM 4-hydroxy-benzeneethanol had little effect on J2 nematode survival. However, exposure to 1.2 mM or a higher concentration resulted in slow-moving J2 nematodes with abnormally stretched bodies. Higher 4-hydroxy-benzeneethanol concentrations (9.8 and 19.2 mM) were lethal to RKNs to some extent, with death rates greater than 50% at 48 h (Fig 6).

## Discussion

RKN infections represent a serious obstacles for continuous cropping in vegetable production facilities [28]. The high toxicity and chemical residues associated with previously developed RKN control measures have hindered the development of environmentally friendly vegetable production systems. Thus, establishment of novel disease management strategies is important for healthy vegetable production. Companion planting can improve the temporal and spatial efficiency of crop production, suppress weed growth and the spread of diseases and insect pests, decrease the need for chemical inputs, and maintain an appropriate ecological balance [29, 30]. In the present study, we observed that the inclusion of Welsh onion as a companion plant decreased RKN-induced damage to cucumber plants, which had fewer root knots and egg masses on the root system (Fig 1). These results reflect that Welsh onion is not a host for RKNs and may be allelopathic to RKNs. Previous studies also report that the use of companion plants, including marigold (*Tagetes patula* L.), basil (*Ocimum basilicum* L.), lettuce (*Lactuca sativa* L.), and white mustard (*Sinapis alba* L.), in greenhouse cultivation of tomato suppress the development of *Meloidogyne* species [31]. These findings reveal that companion planting may provide a method to prevent and control RKN infection.

To examine the mechanism by which Welsh onion inhibits RKNs in cucumbers, we tested the allelopathic effectiveness of Welsh onion root exudates extracted by ethyl ether, ethyl acetate, chloroform, and *n*-butanol. Increasing concentrations of the four root extracts could inhibit the hatching of RKN eggs (Fig 2), implying that Welsh onion root exudates contain various substances that are inhibitory to the hatchability of RKN eggs. This inhibition exhibited an obvious concentration effect, with high concentrations decreasing egg hatchability to less than 50%. Notably, even low concentrations of the *n*-butanol extract significantly decreased RKN egg hatchability (Fig 2C). This result indicated that the *n*-butanol extract must contain compounds that effectively inhibit the hatching of RKN eggs. Analysis by GC-MS identified the most abundant components of the *n*-butanol extract as esters and alcohols, of which the most dominant component was 4-hydroxy-benzeneethanol (50.35%).

Experimental treatments showed that exogenous 4-hydroxy-benzeneethanol significantly restricted the hatching of RKN eggs and was lethal to J2 nematodes. Plants have developed three main mechanisms of resistance against RKNs, which are prevention of interaction between RKNs and the roots [32], interference with normal RKN development, and restriction of RKN growth [16]. We believe that Welsh onion, grown as a companion plant, may decrease RKN-induced damage to cucumber through activity of the root exudates by restricting normal RKN development and retarding RKN growth. The present results are consistent with previous findings that alcohol and benzaldehyde prevent RKN infections [33, 34]. Moreover, in previous studies a novel antifungal compound, fistulosin (octadecyl 3-hydroxyindole), was isolated from roots of Welsh onion and showed high activity against *Fusarium oxysporum* and primarily inhibited protein synthesis [35]. We speculate that 4-hydroxy-benzeneethanol may have a similar effect. The underlying mechanism requires investigation in the future.

Welsh onion root exudates consist of a variety of compounds. We cannot exclude the possibility that other compounds in our study, for example hexahydro-3-(2-methylpropyl)-pyrrolo[1,2-a]pyrazine-1,4-dione, *N*-formyl-tyramine, dibutyl phthalate or hexahydro-3-(phenylmethyl)- pyrrolo[1,2-a]pyrazine-1,4-dione, are involved in inhibition of RKN activity and egg hatchability. It is likely that the exudate compounds may have a complex interrelationship on resistance against RKNs and additional research into their biological functions is required.

The present results provide insight into the role of Welsh onion root exudates on increasing the resistance of cucumber plants to *M*. *incognita*. In addition, the study makes a valuable contribution to the potential use of Welsh onion as a companion plant to protect cucumber plants from RKN infection. We identified 4-hydroxy-benzeneethanol as a major component of Welsh onion root exudates that inhibits the activity of J2 nematodes and decreased the hatchability of RKN eggs. The identification of 4-hydroxy-benzeneethanol as a natural nematicide from Welsh onion root exudates may lay a foundation for development of natural nematicide products.

## Supporting information

S1 Fig Schematic diagrams of the cultivation patterns. A, cucumber plants cultivated in monoculture (with 40 cm spacing between plants; CK). B, Cucumber plants grown with Welsh onion as companion plants (with 8 cm spacing between cucumber and welsh onion plants; 40 cm spacing between cucumber plants T).
(DOC)

S2 Fig Schematic diagram of the Welsh root exudates extraction device. (DOC)

S3 Fig GC-MS spectrum for the other three extracts of Welsh onion root exudates. (A) chloroform extract, (B) ethyl ether extract, and (C) ethyl acetate extract.
(DOC)

S1 Table The main compositions of Welsh onion root exudates extracted with chloroform. (DOC)

S2 Table The main compositions of Welsh onion root exudates extracted with ethyl ether. (DOC)

S3 Table The main compositions of Welsh onion root exudates extracted with ethyl acetate. (DOC)
