# Supplementary figures and images for: Inhibitory effects of components from root exudates of Welsh onion against root knot nematodes
Source: PLoS One. 2018 Jul 30;13(7):e0201471. doi: 10.1371/journal.pone.0201471 (PMC6066241; doi:10.1371/journal.pone.0201471)

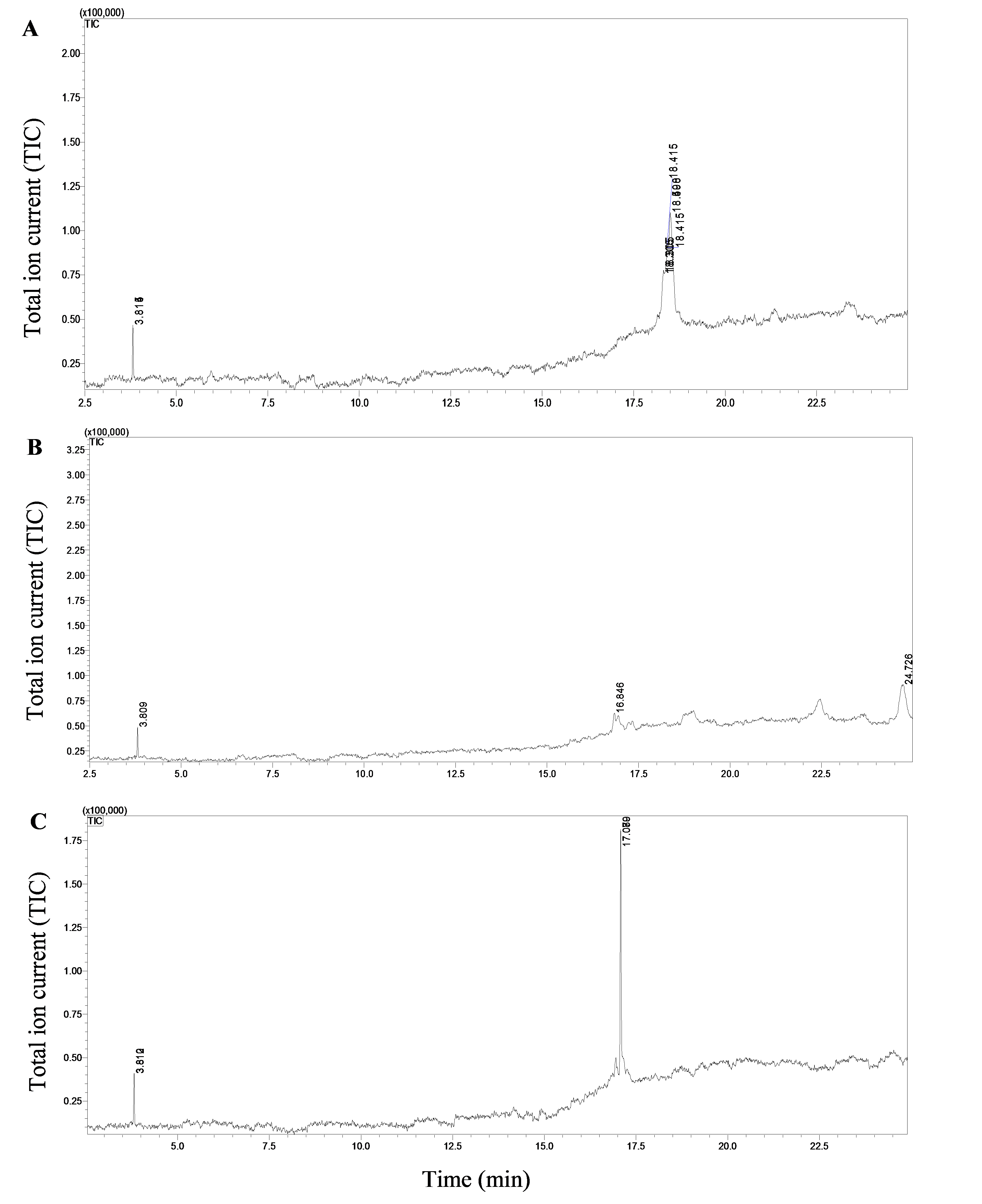

Supplement: S3 Fig — (A) chloroform extract, (B) ethyl ether extract, and (C) ethyl acetate extract. (DOC) [file pone.0201471.s003.doc]
