# Supplementary material for: Inhibitory effects of components from root exudates of Welsh onion against root knot nematodes
Source: PLoS One. 2018 Jul 30;13(7):e0201471. doi: 10.1371/journal.pone.0201471 (PMC6066241; doi:10.1371/journal.pone.0201471)
Supplement: S1 Table — (DOC) [file pone.0201471.s004.doc]

| **Name** | **Chemical structure** | **Percentage composition**  **(%)** | **Similitude index**  **(%)** | **Retention time**  **(min)** |
| --- | --- | --- | --- | --- |
| 4-hydroxy-4-methyl-2-Pentanone | 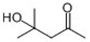 | 6.25 | 94 | 3.814 |
| hexahydro-3-(phenylmethyl)- Pyrrolo[1,2-a]pyrazine-1,4-dione | 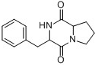 | 23.77 | 88 | 18.305 |
|  |  | 48.71 | 91 | 18.498 |
| 10-dihydro-12'-hydroxy-2'-methyl-5'-(phenylmethyl)-, (5'.alpha.,10.alpha.)- Ergotaman-3',6',18-trione | 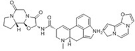 | 21.27 | 61 | 18.415 |
