# Supplementary material for: Inhibitory effects of components from root exudates of Welsh onion against root knot nematodes
Source: PLoS One. 2018 Jul 30;13(7):e0201471. doi: 10.1371/journal.pone.0201471 (PMC6066241; doi:10.1371/journal.pone.0201471)
Supplement: S2 Table — (DOC) [file pone.0201471.s005.doc]

| **Name** | **Chemical structure** | **Percentage composition**  **(%)** | **Similitude index**  **(%)** | **Retention time**  **(min)** |
| --- | --- | --- | --- | --- |
| 2-methyl-2-Hexanol | 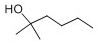 | 18.47 | 89 | 3.810 |
| 1-methyl-9H-Pyrido[3,4-b]indole | 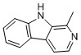 | 13.63 | 89 | 16.845 |
| N-formyl- Tyramine | 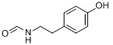 | 67.9 | 86 | 24.725 |
