# Supplementary material for: Inhibitory effects of components from root exudates of Welsh onion against root knot nematodes
Source: PLoS One. 2018 Jul 30;13(7):e0201471. doi: 10.1371/journal.pone.0201471 (PMC6066241; doi:10.1371/journal.pone.0201471)
Supplement: S3 Table — (DOC) [file pone.0201471.s006.doc]

| **Name** | **Chemical structure** | **Percentage composition**  **(%)** | **Similitude index**  **(%)** | **Retention time**  **(min)** |
| --- | --- | --- | --- | --- |
| 4-hydroxy-4-methyl-2-Pentanone | 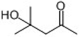 | 15.27 | 94 | 3.810 |
| Dibutyl phthalate | 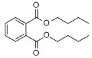 | 84.73 | 98 | 17.079 |
