# Supplementary material for: Inhibitory effects of components from root exudates of Welsh onion against root knot nematodes
Source: PLoS One. 2018 Jul 30;13(7):e0201471. doi: 10.1371/journal.pone.0201471 (PMC6066241; doi:10.1371/journal.pone.0201471)
Supplement: Supplementary file 5 [file pone.0201471.s001.doc]

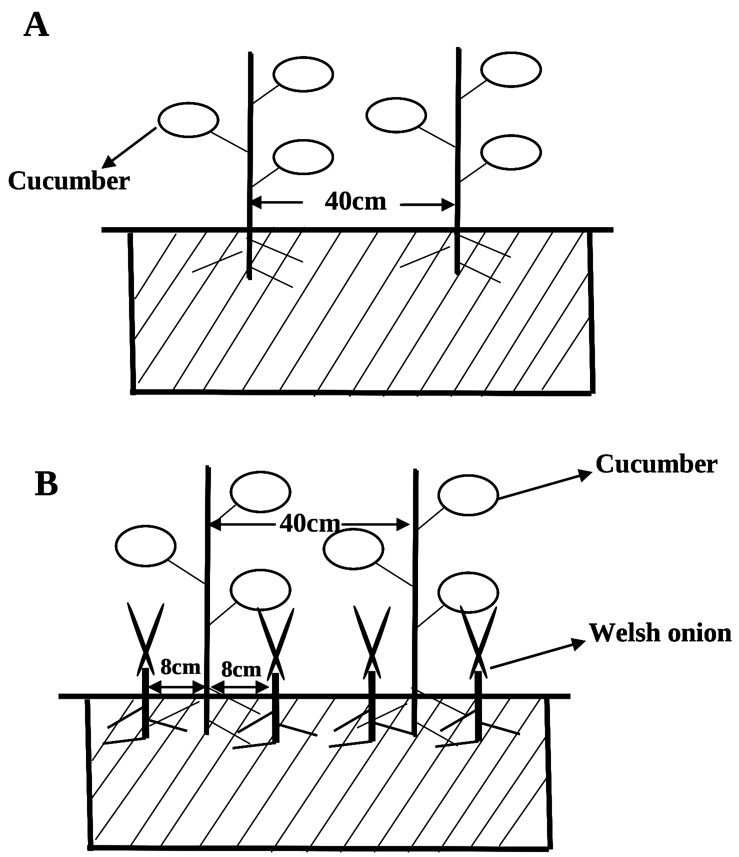
A
Cucumber
40cm
B
Cucumber
40cm
Welsh onion
8cm
8cm
