# Supplementary material for: Inhibitory effects of components from root exudates of Welsh onion against root knot nematodes
Source: PLoS One. 2018 Jul 30;13(7):e0201471. doi: 10.1371/journal.pone.0201471 (PMC6066241; doi:10.1371/journal.pone.0201471)
Supplement: Supplementary file 6 [file pone.0201471.s002.doc]

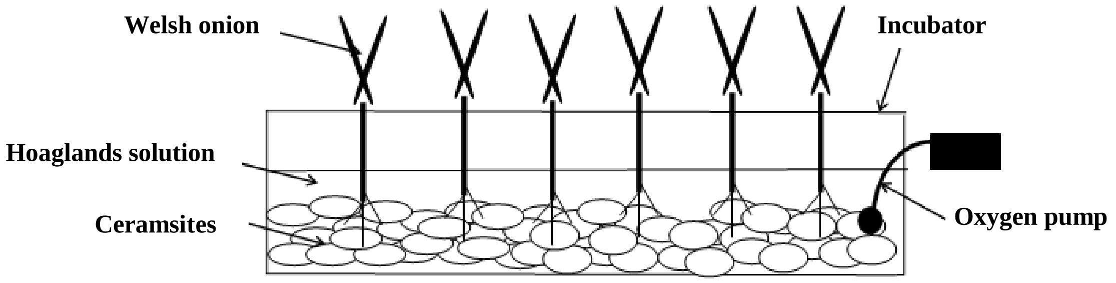
Welsh onion
Incubator
Hoaglands solution
Ceramsites
Oxygen pump
